# Non-neuronal cell-derived acetylcholine, a key modulator of the vascular endothelial function in health and disease

**DOI:** 10.3389/fcvm.2024.1388528

**Published:** 2024-05-15

**Authors:** Takashi Sonobe, Yoshihiko Kakinuma

**Affiliations:** Department of Bioregulatory Science, Graduate School of Medicine, Nippon Medical School, Tokyo, Japan

**Keywords:** ACh, endothelium-dependent vasodilation, the non-neuronal cholinergic system, cardiovascular disease, exercise training

## Abstract

Vascular endothelial cells play an important role in regulating peripheral circulation by modulating arterial tone in the microvasculature. Elevated intracellular Ca^2+^ levels are required in endothelial cells to induce smooth muscle relaxation via endothelium-dependent mechanisms such as nitric oxide production, prostacyclin, and endothelial cell hyperpolarization. It is well established that exogenous administration of acetylcholine can increase intracellular Ca^2+^ concentrations, followed by endothelium-dependent vasodilation. Although endogenous acetylcholine's regulation of vascular tone remains debatable, recent studies have reported that endogenously derived acetylcholine, but not neuronal cell-derived acetylcholine, is a key modulator of endothelial cell function. In this minireview, we summarize the current knowledge of the non-neuronal cholinergic system (NNCS) in vascular function, particularly vascular endothelial cell function, which contributes to blood pressure regulation. We also discuss the possible pathophysiological impact of endothelial NNCS, which may induce the development of vascular diseases due to endothelial dysfunction, and the potential of endothelial NNCS as a novel therapeutic target for endothelial dysfunction in the early stages of metabolic syndrome, diabetes, and hypertension.

## 1 Introduction

Vascular endothelial cells, the key players in maintaining cardiovascular homeostasis, actively modulate the arterial tone in the microvasculature. Our modern lifestyles, often characterized by sedentary behavior, are strongly linked to the development of endothelial dysfunction, a significant contributing factor (1–3). This dysfunction has emerged as a fundamental factor in the pathogenesis of cardiovascular diseases and metabolic complications, including hypertension, obesity, and diabetes (4–6). Various factors, such as hypertension, hyperlipidemia, obesity, diabetes, and aging, induce excess shear stress, oxidative stress, and local inflammation in the vascular endothelial cells, leading to endothelial cell damage. In experimental and clinical studies, endothelial dysfunction is typically characterized by impaired endothelium-dependent vascular relaxation. The physiological response of endothelial cells to exogenous acetylcholine (ACh) is vasodilation (7), which is markedly attenuated in individuals with hypertension (4) and diabetes (8). Reduced responsiveness to ACh is frequently used as a marker of endothelial dysfunction (9, 10). Recent studies have highlighted the importance of “endogenous” ACh, whose origin differs from the classical neurotransmitter ACh in the nervous system (11, 12). The non-neuronal cholinergic system (NNCS) is comprised of various cell types, including immune (13), myocardial (14, 15), and endothelial cells (16), all of which can synthesize and respond to ACh. Endothelial cells respond to exogenously administered ACh to initiate vasodilator signaling. Endothelial function can be modulated by endothelial cells by synthesizing and releasing ACh in an autocrine manner. Considering this, a notable correlation may exist between endothelium-derived ACh and the impaired modulation of endothelial function in the development of cardiovascular disease.

This minireview provides an overview of the roles of non-neuronal cell-derived ACh in regulating vascular endothelial function under physiological and pathophysiological conditions. In particular, we focused on NNCS in endothelial cells, which affect arterial tone. In addition, we discuss the potential therapeutic implications of NNCS function, emphasizing hypertension. One of the main objectives of the current review is to provide additional perspectives on strategies focusing on endothelium-derived ACh to promote vascular health and reduce the risk of developing cardiovascular diseases.

## 2 Effects of ACh on vascular function

The effect of ACh on intact endothelial blood vessels has been well-established since Furchgott’s seminal experiments indicated endothelium-dependent vasodilation (7). Exogenously applied ACh is frequently used to explore endothelial function. ACh administration triggers intracellular Ca^2+^-dependent signaling pathways in vascular endothelial cells (17). This process involves activating the M_3_ muscarinic ACh receptor on endothelial cell membranes (18). Activation of the M_3_ receptor triggers the inositol triphosphate (IP_3_) signaling pathway, resulting in intracellular Ca^2+^ transients from intracellular Ca^2+^ stores and the subsequent production of nitric oxide (NO) (17, 19). ACh-induced endothelium-dependent Ca^2+^ signaling not only activates NO production via endothelial NO synthase (eNOS) but also activates cyclooxygenase, which mediates prostacyclin (PGI_2_) (20) and opens Ca^2+^-activated K^+^ channels (in particular small-conductance, SK3, and intermediate-conductance, IK1), which induces endothelial-derived hyperpolarization (EDH) (21, 22). These three components (i.e., NO, PGI_2_, and EDH) involved in ACh-dependent vasodilation are used to estimate how the endothelial cell capacity is balanced between healthy and impaired modifications of peripheral artery beds (23, 24) given that the magnitude of the contribution of NO, PGI_2_, and EDH differs depending on the artery size (25) as well as the condition of health or disease. For example, exercise training enhances ACh-dependent endothelium-dependent vasodilation under healthy conditions in rats (26, 27). A NO-dependent mechanism mediates training-induced endothelial cell adaptation (26, 28). Furthermore, exercise training has been shown to upregulate NO-dependent and prostacyclin (29)- and EDH (30, 31)-dependent mechanisms. Meanwhile, it is well-known that ACh-dependent vasodilation is substantially altered following endothelial dysfunction induced by hypertension (32), obesity (33), and diabetes (9, 10, 31). Altered ACh-dependent vasodilation may be improved by exercise training, potentially maintaining endothelial cell homeostasis (28). We recently demonstrated that low-intensity exercise training could improve endothelial function in rats with obesity and type 2 diabetes by increasing the contribution of EDH to hind limb arterioles (10). These findings indicate an association between homeostasis of endothelial function and the ACh-dependent signaling pathway in endothelial cells.

## 3 Endogenous source of non-neuronal ACh

Neurons, particularly cholinergic neurons, are the primary source of ACh in the central and peripheral nervous systems. ACh is synthesized, stored in vesicles, and released into the synapses upon neuronal stimulation. As a substrate for ACh synthesis, choline is taken up from the extracellular environment by the high-affinity choline transporter (CHT) (34). Choline acetyltransferase (ChAT), a key enzyme in ACh synthesis, catalyzes the transfer of an acetyl group from coenzyme acetyl-CoA to choline. Vesicular ACh transporter (VAChT) is a member of the proteins constituting the cholinergic system. The VAChT is responsible for loading ACh into secretory vesicles. Acetylcholinesterase (AChE) is responsible for ACh degradation. Experimental studies conducted over the last two decades have revealed that ACh is synthesized and released by various cell types that possess the enzymes and proteins necessary for establishing the local cholinergic system (12). For instance, cardiomyocytes have been found to express these enzymes and proteins, suggesting a role for endogenous ACh in modulating cardiac function independent of neuronal input (14, 15). Notably, ChAT overexpression in murine cardiomyocytes enhances the integrity of brain endothelial function (35). This upregulation of the cardiac cholinergic system, that is, increased ACh synthesis in the heart, does not directly interact with endothelial cells via M_3_ receptors but may activate ascending vagus nerve signaling (35, 36).

Two major sources of non-neuronal ACh directly affect endothelial function: lymphocytes and endothelial cells. Endothelial cells are discussed in a separate section.

### 3.1 Lymphocytes

Given that ACh can be measured in the human blood (13), Kawashima et al. explored the origin of ACh and identified T lymphocytes as the source of circulating ACh (13, 37). Lymphocytes that express ChAT have been characterized as a subset of T-helper cells, such as CD4^+^, CD44^hi^, and CD62 L^lo^ (38). Lymphocytes regulate blood pressure via endothelium-dependent mechanisms by increasing the bioavailability of ACh in the blood. In a small animal experiment, infusion of ChAT-overexpressing Jurkat *T* cells reduced the mean arterial blood pressure in mice (38). Moreover, human ChAT-positive T cells have been shown to release ACh and promote arterial relaxation through cholinergic mechanisms (39, 40). Although *T*-cell-derived ACh can induce endothelium- and NO-dependent vasodilation, it is unlikely that *T*-cells are constantly activated to release ACh to modulate endothelial function and blood pressure under normal physiological conditions. Additional investigations are needed to establish the effects of *T* cell-derived ACh on cardiovascular diseases, particularly inflammation-related vascular dysfunction (40, 41).

## 4 ACh synthesized in endothelial cells

The ability of ACh to stimulate endothelium-dependent vasodilation has been demonstrated in several vascular beds (9, 10, 42). The origin of ACh present *in vivo*, particularly in the bloodstream, acting on vascular endothelial cells, has long been debated (43–45). First, the parasympathetic nerve ending is a typical source of ACh; however, few blood vessels are innervated by parasympathetic nerves (46). Second, autonomic nerves are usually located on the adventitial side of the blood vessel wall, and any ACh released from parasympathetic nerve endings faces the basal lamina barrier before reaching the endothelial cell membrane, where the M_3_ ACh receptors are localized. Third, in the 1930s, researchers identified the presence of ACh in ox blood (47, 48), and subsequent studies also reported the presence of ACh in the blood, as discussed in the previous section (13). Even if physiologically sufficient concentrations of ACh are present in the blood, it is unlikely that endothelial cells will be persistently activated by circulating ACh because of its inactivation by cholinesterase, which rapidly deactivates ACh into acetate and choline (49). ACh spillover is an intriguing idea in which ACh is released via activation of the motor nerve and spills over onto muscarinic receptors on the arteries of skeletal muscles, resulting in vasodilatation (50). This is reasonable because active skeletal muscles require adequate blood supply, which the peripheral vascular tone regulates.

Milner et al. reported that endothelial cells can produce and release ACh (51, 52). Using a modified chemiluminescence assay, the authors detected the ACh content in the perfusate of rat Langendorff heart preparations during hypoxia (51). In a subsequent study, they also demonstrated a substantial increase in ACh content in the perfusate from cultured umbilical vein endothelial cells during a high-flow perfusion period under shear stress conditions (52). The presence of ACh derived from endothelial cells has also been documented by Kawashima et al., who demonstrated its presence in the culture supernatant of bovine arterial endothelial cells using a radioimmunoassay (53). The presence of endothelial ACh was confirmed by electrochemical detection combined with high-performance liquid chromatography, which identified an ACh-derived peak in human umbilical vein endothelial cells (54).

Endothelial cells express ChAT, similar to other tissues, such as immune cells, in which NNCS exists. In the 1980s, Parnavelas et al. demonstrated that ChAT immunoreactivity is localized in the endothelial cells of capillaries and small vessels in the rat brain (43). Notably, the authors also suggested the synthesis and release of ACh from endothelial cells. Endothelial cells also express other essential components of the cholinergic system, including VAChT (55), CHT (56), and AChE (57, 58), which can develop self-containing NNCS in endothelial cells. Increasing evidence has indicated that vascular endothelial cells are an important source of ACh in the NNCS, thereby establishing an autocrine regulatory loop for endothelial function via the M_3_ receptor in the vascular microenvironment (16). The local production of ACh by endothelial cells contributes to fine-tuning vascular tone under physiological conditions. Wilson et al. suggested that autocrine-like regulation of endothelial function mediated by ACh is required to facilitate flow-mediated vasodilation via shear stress-dependent activation of the intracellular Ca^2+^ signaling pathway in endothelial cells (16). The autocrine-like modulation of endothelial function via local endothelial ACh may support the role of small peripheral arteries, which necessitate local EDH to induce ascending vasodilation (59). This mechanism may share the signaling pathways of mechanosensitive modulation of endothelial function, which also activates intracellular Ca^2+^ signaling pathways in response to shear stress (60–62). Although the mechanisms by which ACh release is activated by changes in flow (shear stress) remain unclear, local ACh signaling potentially influences exercise-induced hyperemia triggered by local vasodilation and vasodilation during skeletal muscle contraction (59).

## 5 Endothelial ACh in cardiovascular disease

Arterial vasodilation caused by exogenous ACh administration is impaired in several cardiovascular diseases (63). An impaired vasodilator response is often defined as endothelial dysfunction because an endothelium-dependent mechanism mediates the vasodilator effect of ACh (7). ACh is a pharmacological tool for examining whether endothelial cells respond to external stimuli mediated by ACh receptors. However, recent evidence suggests impaired endothelial function is associated with NNCS dysfunction.

Considering lymphocytes, in which the NNCS is known to exist, levels of ChAT expression and ACh production decreased in spontaneously hypertensive rats (SHR) (64). Similarly, the genetic ablation of ChAT in ChAT-positive T cells results in hypertension in mice, which is attributed to an endothelium-eNOS-dependent mechanism (38). These findings suggest that NNCS lymphocyte dysfunction triggers the development of hypertension in an ACh endothelial axis-dependent manner. Interesting experimental approaches have been employed to overcome hypertension in rodents (65). In mice with angiotensin II-induced hypertension, systemic administration of recombinant ChAT substantially reduced the mean arterial blood pressure. Notably, this ChAT-induced decrease in blood pressure in hypertensive mouse models was reversed by inhibiting NO production, indicating that the effect of exogenous ChAT administration on systemic blood pressure is NO-dependent (65). Different experimental studies have demonstrated that exogenous choline administration delays the progression of hypertension in SHR, presumably by enhancing vagus nerve activity (66). Moreover, serum ACh levels were substantially lower in SHR than in control Wistar Kyoto rats (WKY); choline administration restored these levels. This concept of increasing the bioavailability of circulating ACh, which directly acts on ACh receptors in endothelial cells, may offer a potential strategy to prevent the development of hypertension caused by depletion or dysfunction of the circulating ACh source. The bioavailability of circulating ACh may be clinically relevant because low plasma ACh levels and endothelial dysfunction are associated with increased mortality in critically ill patients with poor cardiovascular outcomes (67–69).

**Figure 1:**
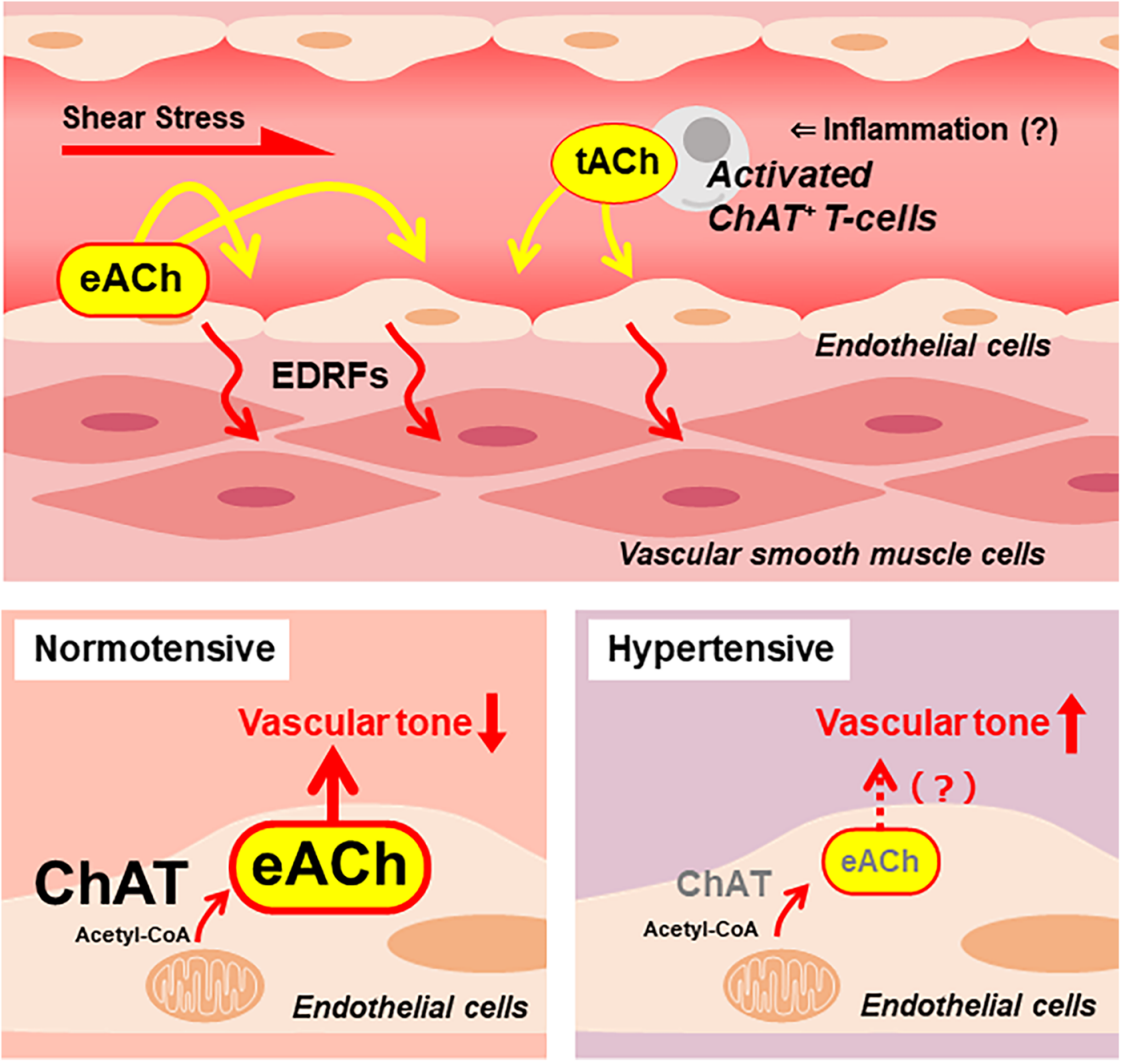
A putative endothelial non-neuronal cholinergic system that regulates endothelium-derived relaxing factors (EDRFs) modulating vascular tone. Increased shear stress stimulates vascular endothelial cells to release ACh. The endothelial cell-derived ACh (eACh) affects the endothelial function in an autocrine manner. Local inflammation recruits activated ChAT-positive *T* cells, which release ACh. The *T* cell-derived ACh (tACh) is also suggested to affect endothelial function. In a healthy, normotensive condition, ChAT in endothelial cells contributes to the synthesis of eACh. It is hypothesized that adequate storage and release of eACh maintains basal vascular tone. Meanwhile, endothelial ChAT decreases in the condition of hypertension. Thus, it causes decreased eACh synthesis that results in increased vascular tone.

As discussed in the previous section, vascular endothelial cells are an important source of ACh. Therefore, a new hypothesis was proposed: endothelial NNCS dysfunction is responsible for endothelial dysfunction. Although there is limited experimental evidence supporting this hypothesis, a study by Zou et al. revealed that the expression of VAChT protein, an NNCS component, was markedly lower in the aortic ring of SHR than in the aortic ring of control WKY (70). Additionally, the concentration of ACh in the aortic ring was lower in SHR than in WKY after choline incubation. These findings suggest that the decreased synthesis and release of ACh from endothelial cells may impair endothelial function, impairing arterial tone regulation, thereby contributing to hypertension (Figure 1). Additionally, evidence not directly related to the endothelium revealed that NNCS in the heart is downregulated in mice and humans with obese type 2 diabetes (71). Considering these findings, NNCS may be down-regulated in the endothelial cells under diabetic conditions. Further studies are required to clarify the relationship between endothelial NNCS and diabetes-induced microvascular dysfunction.

The clearly defined clinical manifestations of endothelial NNCS dysfunction in humans remain poorly understood. Instead, the implications of endothelial dysfunction have been frequently discussed in the context of its role in various clinically relevant cardiovascular diseases, including hypertension and diabetic microangiopathy. Future studies are needed to shed light on endothelial NNCS as potential therapeutic targets for these diseases.

## 6 Conclusion

In conclusion, understanding NNCS in the vascular system is increasingly important as it may contribute to the development of cardiovascular disease by modulating arterial tone. Recent findings indicate that endogenous ACh is pivotal in regulating endothelial signaling mechanisms. Investigating the pathophysiological significance of endothelial NNCS dysfunction may improve our understanding of the early stages of vascular diseases caused by endothelial dysfunction. These findings suggest that endothelial NNCS may be a potential therapeutic target for conditions such as metabolic syndrome, diabetes, and hypertension and may lead to the development of novel therapeutic strategies.
